# Large-scale global molecular epidemiology of antibiotic resistance determinants in Streptococcus pneumoniae

**DOI:** 10.1099/mgen.0.001444

**Published:** 2025-07-02

**Authors:** Kazi Shefaul Mulk Shawrob, Achal Dhariwal, Gabriela Salvadori, Rebecca A. Gladstone, Roger Junges

**Affiliations:** 1 Institute of Oral Biology, Faculty of Dentistry, University of Oslo, Oslo, Norway; 2 The Intervention Centre, Oslo University Hospital, Oslo, Norway; 3 Department of Biostatistics, Faculty of Medicine, University of Oslo, Oslo, Norway

**Keywords:** antimicrobial resistance, macrolides, molecular epidemiology, multiple drug resistance, *Streptococcus pneumoniae*, surveillance, whole-genome sequencing

## Abstract

*Streptococcus pneumoniae* is a leading pathogen in terms of deaths attributable to or associated with antimicrobial resistance globally. Thus, monitoring antibiotic resistance determinants constitutes a key aspect of surveillance efforts for this microbe. Leveraging publicly available whole-genome sequencing (WGS) data, we aimed to investigate the presence and distribution patterns of antibiotic resistance determinants in *S. pneumoniae* with a focus on multidrug resistance (MDR) and serotype distribution. Metadata and genomes were obtained from the National Center for Biotechnology Information Pathogen Detection database. Curation and harmonization were performed in R and SPSS. Data on resistance patterns were defined according to AMRFinderPlus, and a combination of prediction tools was employed for *in silico* serotyping. Analyses involved 75,161 genomes totalling 122,673 gene/allele counts from 14 antibiotic classes. MDR was observed in 16.7% of isolates, with the highest increasing rates in Asia and South America. Within antibiotic classes, an increase in macrolide resistance genes was highlighted, particularly in the proportion of genomes presenting *mef(A*)/*msr(D*). Over a third of isolates with serotypes 19F, 23F, 15A, 6B and 19A showed MDR. We further observed the highest significant increases in the presence of resistance in 33F, 22F, 10A and 23A. Serotype 13, not included in any vaccine formulation, presented high MDR rates with a strong increasing trend. The findings of this study highlight variations in resistance determinants globally and across serotypes over time. Collectively, these data underscore the added value of utilizing public WGS data to investigate the effectiveness and repercussions of treatment and vaccination strategies on managing antibiotic resistance.

**Impact Statement**

Antibiotic resistance in the major human pathogen *Streptococcus pneumoniae* creates challenges for treatment, causing increased rates of morbidity and fatality globally. In this study, we have leveraged over 75,000 publicly available pneumococcal whole-genome sequences with the aim of surveying the patterns and proportion of antimicrobial resistance determinants. Even though multidrug resistance rates over the years have been seemingly stable globally, increasing trends were observed for genomes isolated from Asia and South America. In line with recent findings, a rise in macrolide resistance was highlighted. Finally, through *in silico* serotype prediction, we were able to identify a series of capsular serotypes that show a concerning increase in the presence of resistance determinants over recent years. This includes serotypes covered and not covered in the most commonly employed vaccines. This study underscores the added value of utilizing public genomic sequences to investigate the effectiveness and repercussions of treatment and vaccination strategies on managing antibiotic resistance in *S. pneumoniae*. It further offers an early warning system for the emergence of new antibiotic-resistant serotypes, strains or clones.

## Data Summary

Data utilized in this study are public and available in the NCBI Pathogen Detection Isolates Browser (https://www.ncbi.nlm.nih.gov/pathogens/) accessed on 12 August 2024. Data were obtained from the National Center for Biotechnology Information and accession numbers for all genomic sequences retrieved are listed in Table S1. Protocols utilized in this study have been detailed and provided in the article and supplementary data files.

## Introduction

*Streptococcus pneumoniae* is a leading cause of non-invasive and invasive disease worldwide, especially in young children and older adults [1]. In 2019, *S. pneumoniae* was one of five pathogens – together with *Staphylococcus aureus*, *Escherichia coli*, *Klebsiella pneumoniae* and *Pseudomonas aeruginosa* – estimated to cause over 4 million deaths worldwide. The impact was particularly severe in children, as *S. pneumoniae* was the pathogen associated with most deaths among children younger than 5 years [2,3]. The development and spread of antimicrobial resistance (AMR) create further challenges, as it leads to longer hospital stays, higher mortality rates and a substantial economic burden [4].

The capsular polysaccharide is the primary virulence determinant in *S. pneumoniae*, with current vaccine strategies being based on protection against a selection of these serotypes. Understanding which serotypes have higher chances of carrying antibiotic-resistant determinants is crucial for clinical treatment as well as informed decisions on vaccine strategy employment and further research development. There are six available pneumococcal conjugate vaccines (PCVs) and one pneumococcal polysaccharide vaccine (PPSV23). These vaccines have helped to reduce the incidence of invasive disease [5]; however, coverage is a challenge [6], and less than a fourth of over 100 serotypes are currently included in these vaccines, which creates risks for the emergence of non-vaccine serotypes [7,8]. As the pneumococcus exhibits high rates of horizontal gene transfer, further risks of serotype switching and vaccine escape are also significant [9]. The emergence of beta-lactam resistance in *S. pneumoniae* in the 80s and 90s led to a higher utilization of other classes of drugs such as macrolides and fluoroquinolones. Recently, the World Health Organization has published the 2024 Bacterial Priority Pathogens List [10], which includes macrolide-resistant *S. pneumoniae*, as a response to the concerning rise in resistance in the species, particularly in low- and middle-income countries and in vulnerable populations [11,12]. Globally, rates of macrolide resistance vary depending on the region, with a wide range of 1–99% [13,15].

Microbial phenotypical testing in the laboratory remains the gold standard for assessment of resistance patterns in pathogens to assist clinical treatment. However, the utilization of whole-genome sequencing (WGS) data can enhance surveillance efforts for AMR, as the detailed information obtained from genomic sequences allows the investigation of specific genes and their alleles, together with the integration of metadata and further virulence markers [16,17]. This strategy allows for the understanding of the genetic distribution of resistance determinants, trends in development, co-occurrence patterns and multidrug resistance (MDR), among others [18,19]. While phenotypic and genomic data integration challenges persist, the benefits of this combined strategy are substantial [19]. In addition, recent studies indicate strong correlations between genotypic inference and phenotypical data [20,21]. With the further advancement of on-site sequencing speeds and machine learning tools for phenotype prediction, genomic assessment might potentially be employed to enable rapid clinical diagnosis and decision-making [22,23]. Regardless, it remains a valuable tool for retrospective analyses.

As the number of genomes available in public repositories rapidly increases, this manifests as an important resource for genomic surveillance. Recent studies have shown the added value of utilizing such data for tracking and surveillance of AMR in probiotic bacteria [24] and in different human and animal pathogens, including *E. coli* [25], *Salmonella* [26], *Campylobacter* spp. [27], *S. aureus* [28] and *Streptococcus agalactiae* [29]. As such, the aims for this study were to leverage pneumococcal genomic data mined from public repositories to investigate the distribution of antimicrobial resistance genes (ARGs), occurrence of MDR and temporal patterns of resistance across serotypes.

## Methods

### Genomic data

Data were obtained from the National Center for Biotechnology Information (NCBI) Pathogen Browser (https://www.ncbi.nlm.nih.gov/pathogens/) on 12 August 2024, which includes genomic sequences and metadata from a variety of sources, including public health surveillance programmes. In the database, AMR gene classification was performed based on the AMRFinderPlus tool (v3.11.26 and v3.12.8) [30]. Reported resistance genes or divergent alleles calling follows the reference gene catalogue for the referred resource tool (https://www.ncbi.nlm.nih.gov/pathogens/refgene/). Furthermore, antimicrobial class and subclass categories that were followed are available on GitHub (https://github.com/evolarjun/amr/wiki/class-subclass). For subsets of analyses, genomic sequences were retrieved utilizing the NCBI Datasets pipeline, and the match data function in R was employed to obtain metadata information based on their respective accession numbers. The high performance computing (HPC) clusters maintained by the University of Oslo IT Department were utilized for parts of the analyses.

### Dataset curation

Data were imported into R (v4.3.2). Curation of metadata was performed with the tidyverse (v2.0.0), dplyr (v1.1.4), stringr (v1.5.1), magrittr (v2.0.3) and countrycode (v1.6.0) packages. Further details on data handling and curation are available in the supplementary material.

### MDR and resistance determinant calling

MDR was defined as isolates with reported resistant genotypes to three or more unique classes. A new MDR classification column was created, and an MDR score was calculated based on previous studies [25,26]. MDR score measures the number of antibiotic classes a genome is predicted to be resistant to, based on ARGs or divergent alleles. This score uses a discrete numerical scale, removing duplicates and counting unique antimicrobial classes. For antibiotic resistance gene calling, NCBI’s AMRFinderPlus [30] criteria were employed with only complete genes being included. The only exception was that we excluded the *pmrA* gene, which composes a transporter system together with *pmrB* that is often associated with resistance to antimicrobial peptides and can also confer resistance to some antibiotics. Its presence is mostly ubiquitous in *S. pneumoniae* and its mode of function differs from conventional ARGs [31,32]; thus, we opted for removal from the dataset. In addition, it is important to note that penicillin-binding proteins (*pbps*) are core genes in streptococci; however, they can exhibit divergent allele variations and combinations conferring resistance to a variety of beta-lactam antibiotics. AMRFinderPlus reports the three main *pbps* when alleles show protein identity below pre-defined thresholds of 99% for *pbp1a*, 99% for *pbp2b* and 98% for *pbp2x*, as compared to the reference gene catalogue.

### Serotype classification and statistical analyses

To investigate the relationship between ARGs and capsules in this collection of isolates, serotyping was performed by retrieving sequences for all genomes linked to an accessioned assembly (*n*=45,376). Details regarding the serotyping strategy are available in the supplementary material. For convenience, the list of serotypes included in each vaccine category is available in Table S2, available in the online Supplementary Material. Cross-tabulations and descriptive analyses were performed using SPSS and R Studio. A two-sided *P* value of <0.05 was considered statistically significant. For temporal trend analysis utilizing MDR score, negative binomial regression or Poisson regression was employed using the MASS package (v7.3–60.0.1), considering count data as a metric and the overdispersion presented in the analysed data. Temporal trend analyses with relative proportions were performed employing quasibinomial regression.

## Results

### Data description

**Fig. 1.**
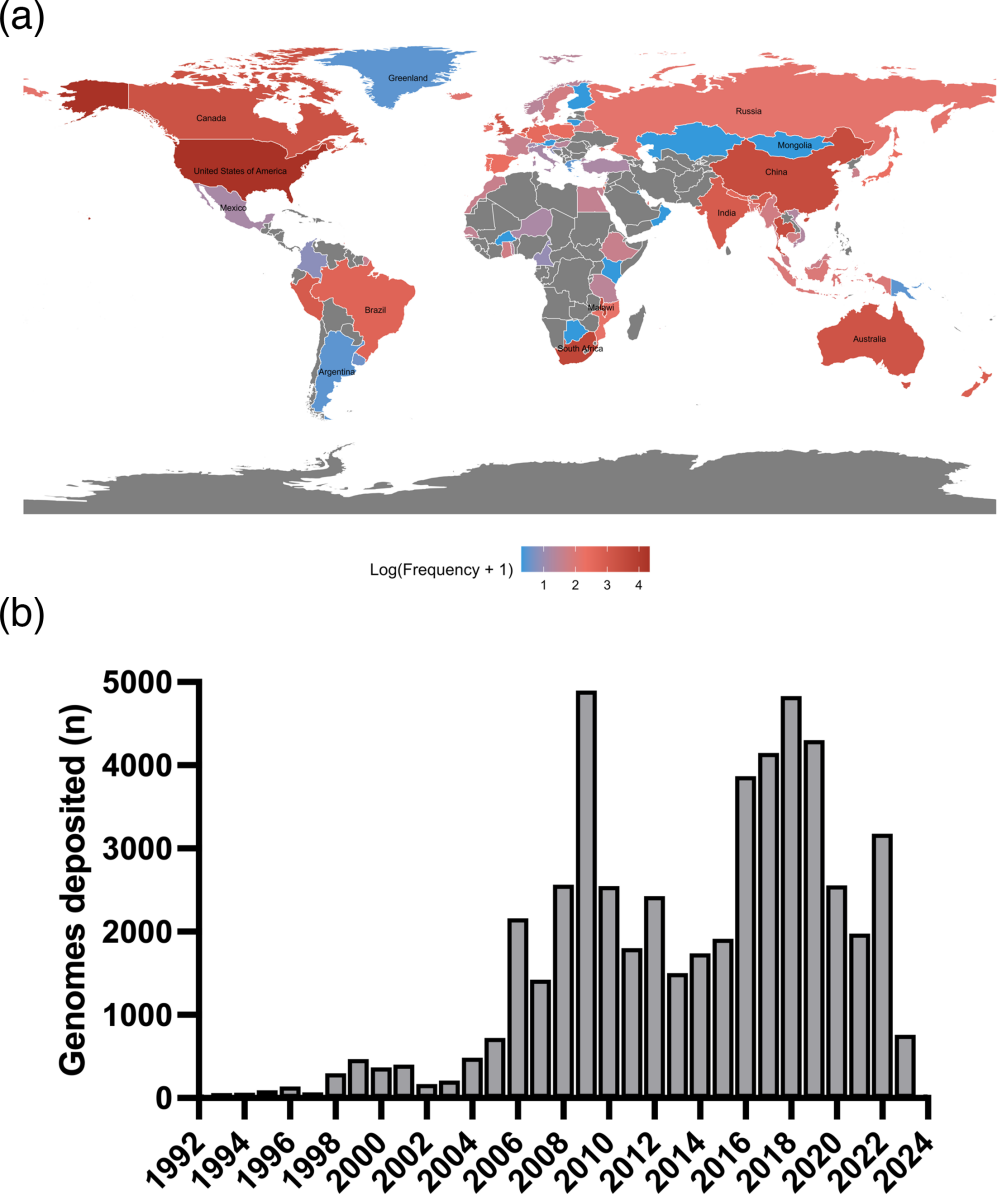
Geographic distribution and year of collection for *S. pneumoniae* isolates. (**a**) Global heatmap of number of isolates per country in logarithmic scale. (**b**) Year of collection with data shown from 1992 and forward, as >40 genomes have been consistently collected since then. Prior to 1992, a total of 209 genomes distributed across ten decades were collected.

Metadata for 75,161 *S*. *pneumoniae* genomes were analysed. Of these, 53,606 genomes presented location information with continent distribution as North America (*n*=23,356; 31.1%), Africa (*n*=11,362; 15.1%), Asia (*n*=9,670; 12.9%), Europe (*n*=5,195; 6.9%), Oceania (*n*=2,377; 3.2%) and South America (*n*=1,646; 2.2%). Fig. 1(a) shows a global heatmap of the number of isolates per country, supplemented by Table S3. The year of collection was available for 51,864 genomes and their distribution is available in Fig. 1(b). The source of collection was most commonly reported as a sterile site (*n*=17,642; 23.5%) and blood (*n*=10,945; 14.6%). Missing information was commonly found in the metadata analyses, as 21,555 (28.7%) presented no location information, 23,297 (31.0%) presented no date for collection and 26,392 (35.1%) listed no isolation source.

### MDR and gene co-occurrence

**Fig. 2.**
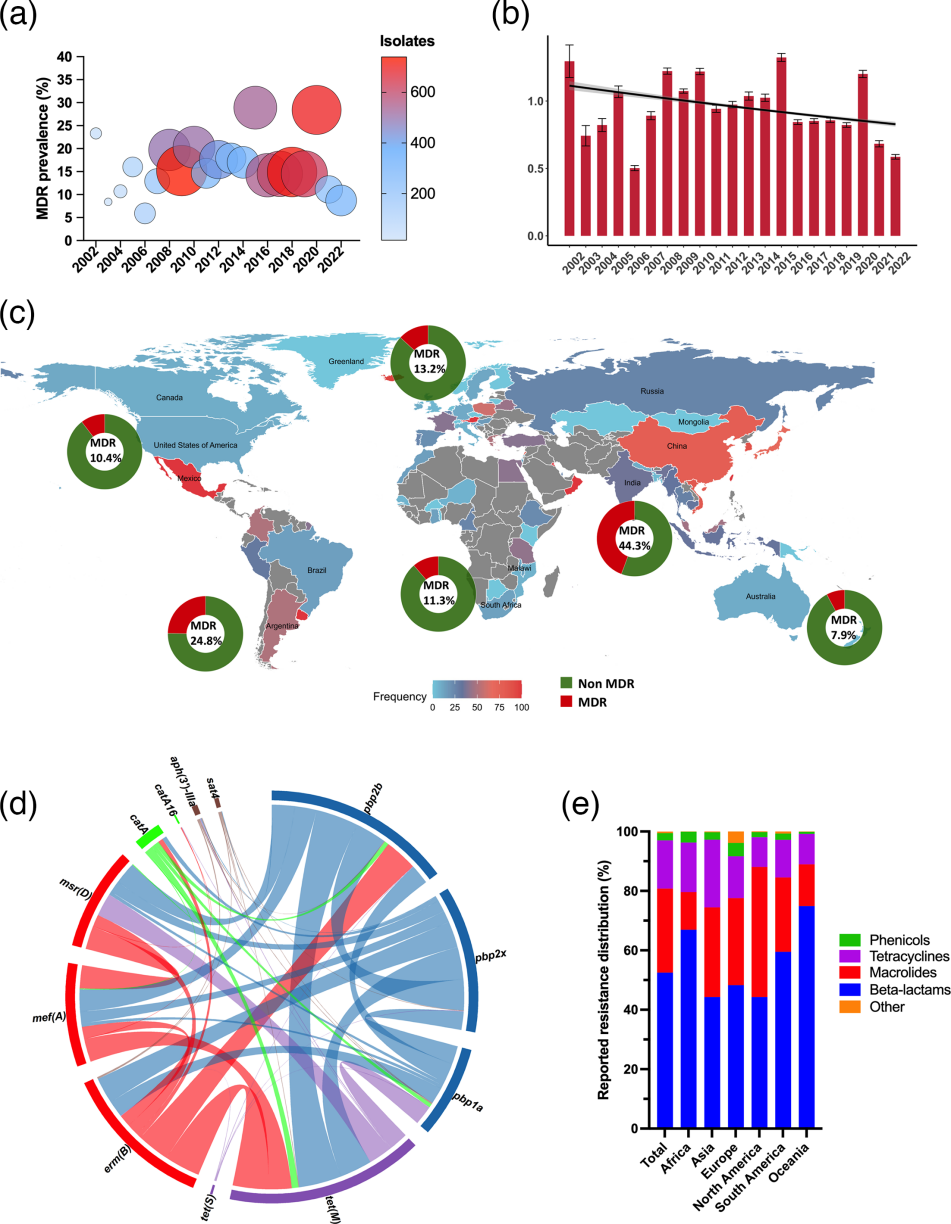
Presence of MDR in *S. pneumoniae* isolates. (**a**) Proportion and number of isolates with MDR by year. (**b**) MDR score over time using negative binomial regression (*P*<0.05). (**c**) Relative presence of MDR per continent in each donut plot and by country according to the colour code. (**d**) Chord diagram of gene co-occurrence in MDR isolates (threshold of 100 occurrences and above), with genes from the same antibiotic class grouped into one colour. (**e**) Distribution of resistance towards the four most frequent classes in the total population and in each continent.

**Fig. 3.**
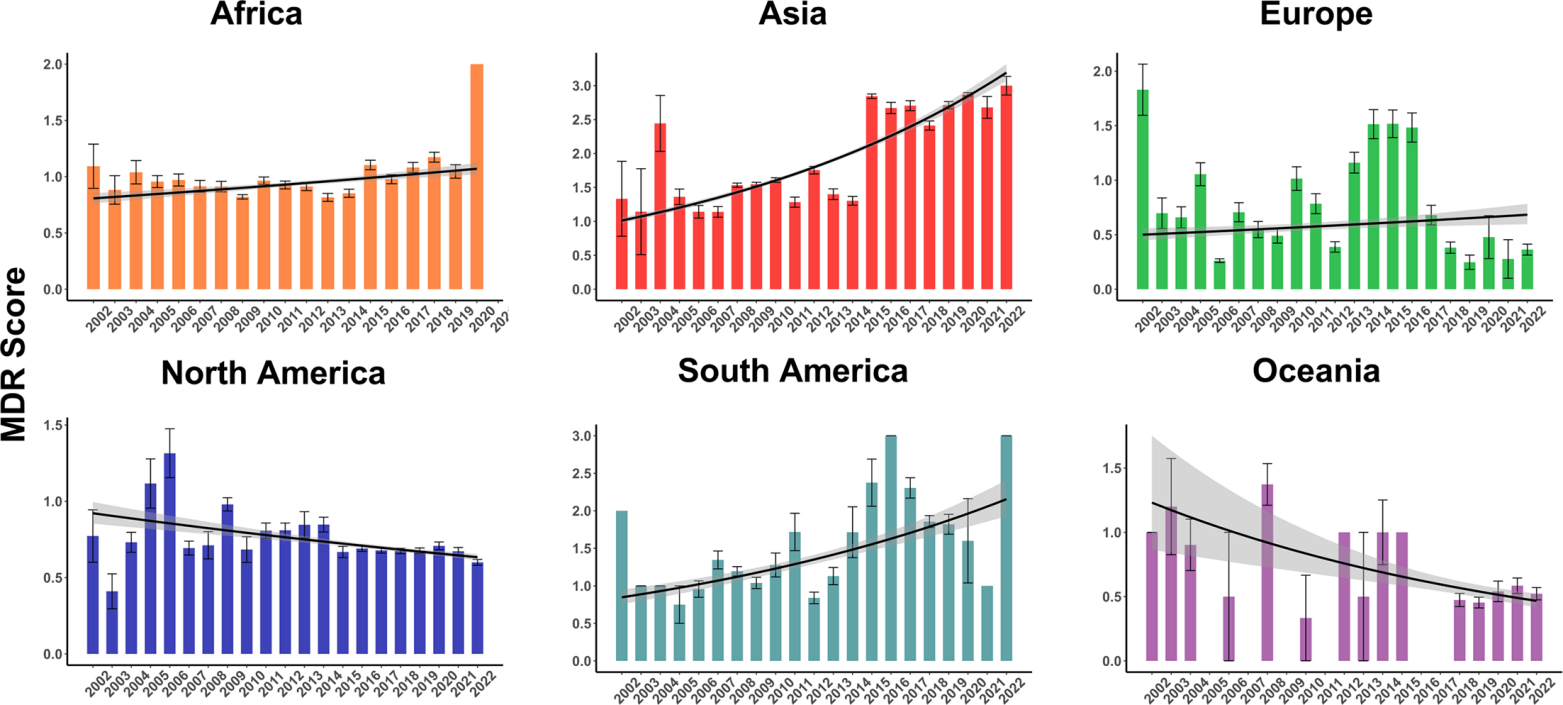
MDR score by year in each continent. Bars represent mean MDR scores with sem, and lines represent the fitted trends derived from negative binomial regression, while the shading represents the 95% confidence intervals. All trends were significant (***, *P*<0.001), with the exception of Europe, where a lower significance level was observed (*, *P*<0.05).

In total, 12,554 genomes (16.7%) presented MDR predicted by the genotype. As such, the average MDR score across all samples was 0.98 with an sem of 0.004. Most genomes presented no ARGs (*n*=35,695), and the maximum resistance determinants observed were in three genomes with resistance to seven antibiotic classes. Distribution over years remained stable, considering all world regions. Fig. 2a shows MDR proportion over the years, while Fig. 2b reveals a declining trend in MDR scores over time. Data for each country and region on MDR are available in Fig. 2c. With data stratified by world region, the strongest increasing trends were observed for Asia and South America in recent years in terms of MDR score (Fig. 3). In MDR isolates, the association between divergent *pbp2bs* and *tet(M*) showed the highest frequency (10,943 co-occurrences). Additionally, *erm(B*) and *tet(M*) co-occurred 9,109 times, while *erm(B*) and divergent *pbp2bs* were found together in 8,346 instances (Fig. 2d).

### Class and gene distribution

**Fig. 4.**
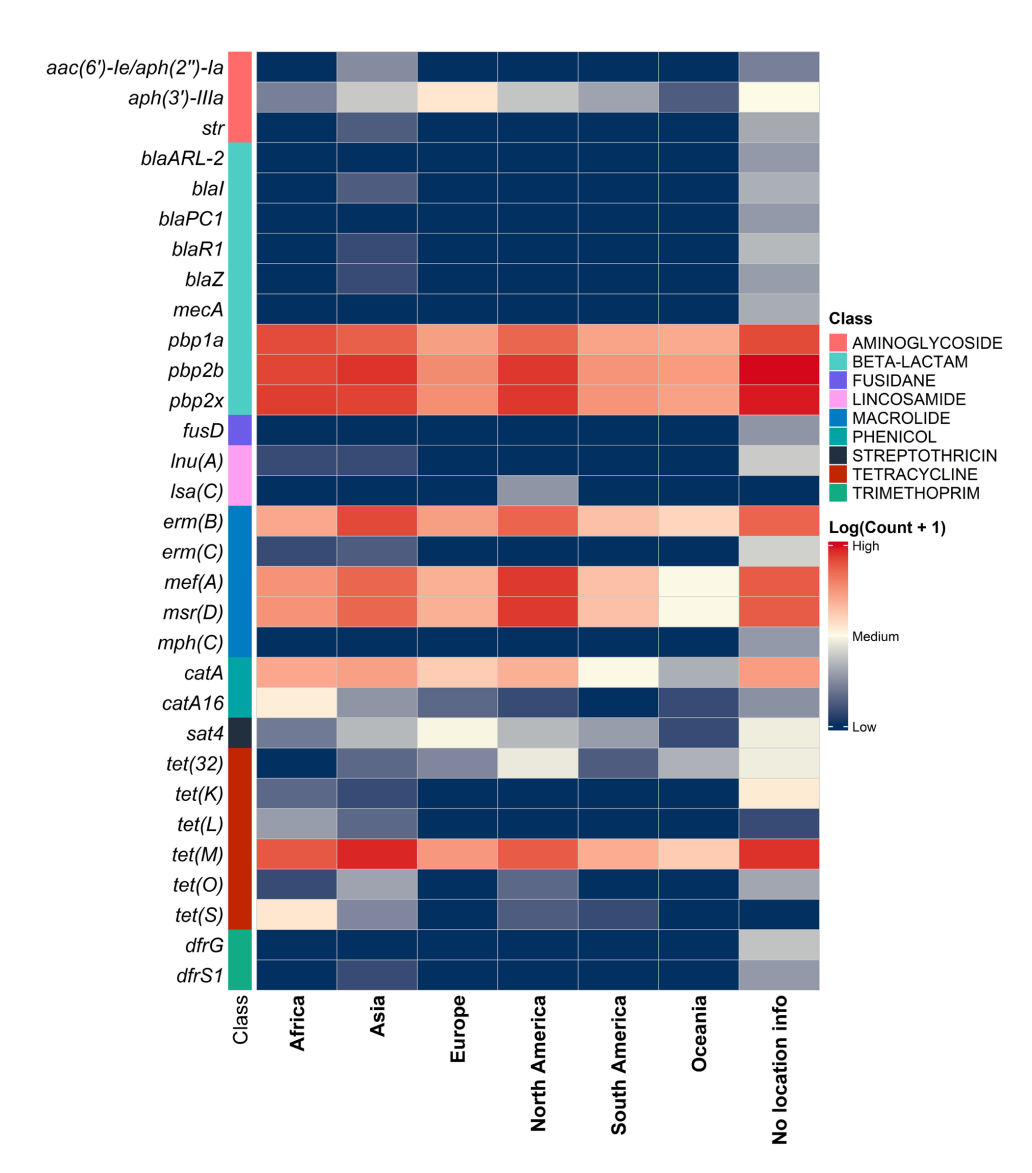
Heatmap showing relative presence of resistance genes and divergent *pbp*s in *S. pneumoniae* genomes grouped by continent. Genes are grouped by colour according to antibiotic class, and determinants with a count below 10 were removed. The complete list of genes is available in Table S4.

**Fig. 5.**
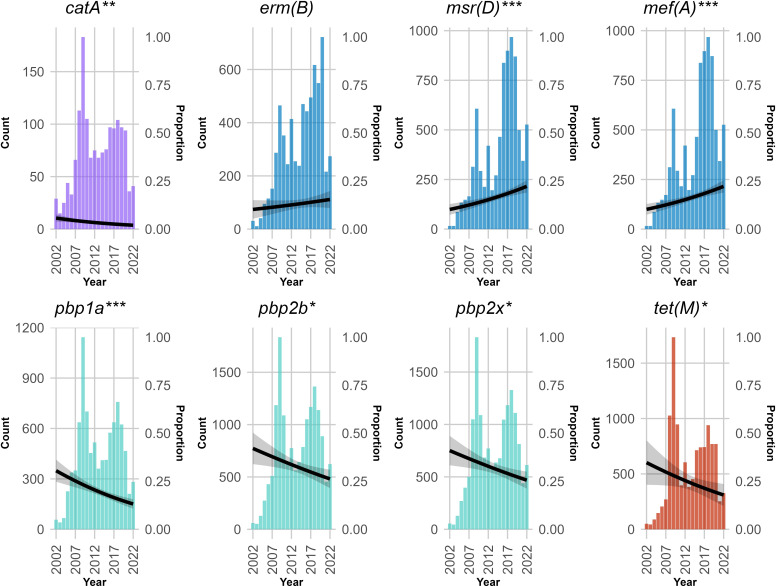
Development over time for the eight most common resistance determinants in *S. pneumoniae* isolates. Colours indicate grouping by antibiotic class. Lines represent the fitted MDR proportion, while the shading represents the 95% confidence intervals. Fitted curves were generated with quasibinomial regression, also indicating statistical significance. *P<0.05, **P<0.01, ***P<0.001.

A total of 72 unique resistance determinants and 122,673 counts of either ARGs or reported resistance alleles were observed. The four antibiotic classes in which resistance was commonly predicted to were beta-lactams (64,427; 52.5% of the total ARGs and 40.9% of genomes containing at least one ARG/divergent allele), macrolides (34,641; 28.2% of the total ARGs and 25.9% of genomes containing at least one ARG), tetracyclines (19,926; 16.2% of the total ARGs and 26.4% of genomes containing at least one ARG) and phenicols (2,986; 2.4% of the total ARGs and 4.0% of genomes containing at least one ARG). Relative distribution of these classes in total and in each continent is available in Fig. 2e. Development trends over the years for the three most common classes in each continent are shown in Fig. S1. The list of ARGs/divergent resistance alleles identified is available in Table S4, and their distribution across continents as a heatmap is shown in Fig. 4. For the eight most frequent resistance determinants, temporal development is visualized in Fig. 5. Over time, the presence of determinants that can confer resistance to beta-lactams and tetracyclines is seemingly decreasing, while an increase in macrolide resistance was observed. For genotype distribution regarding macrolide resistance, higher proportions of *mef(A*) resistance were observed (20.4%) compared to *erm(B*) (14.3%), with 3.6% of genomes presenting dual-genotype resistance. However, this varied across world regions (Table S5). While genomes from Africa and North America showed predominance of the *mef(A*) genotype, the opposite was seen for isolates from Asia, Europe, South America and Oceania. For less frequently present genes, *tet(32*) stood out with 160 occurrences, given that a significant number had been observed from 2017 to 2022 (*n*=67).

### Serotype association with AMR

**Fig. 6.**
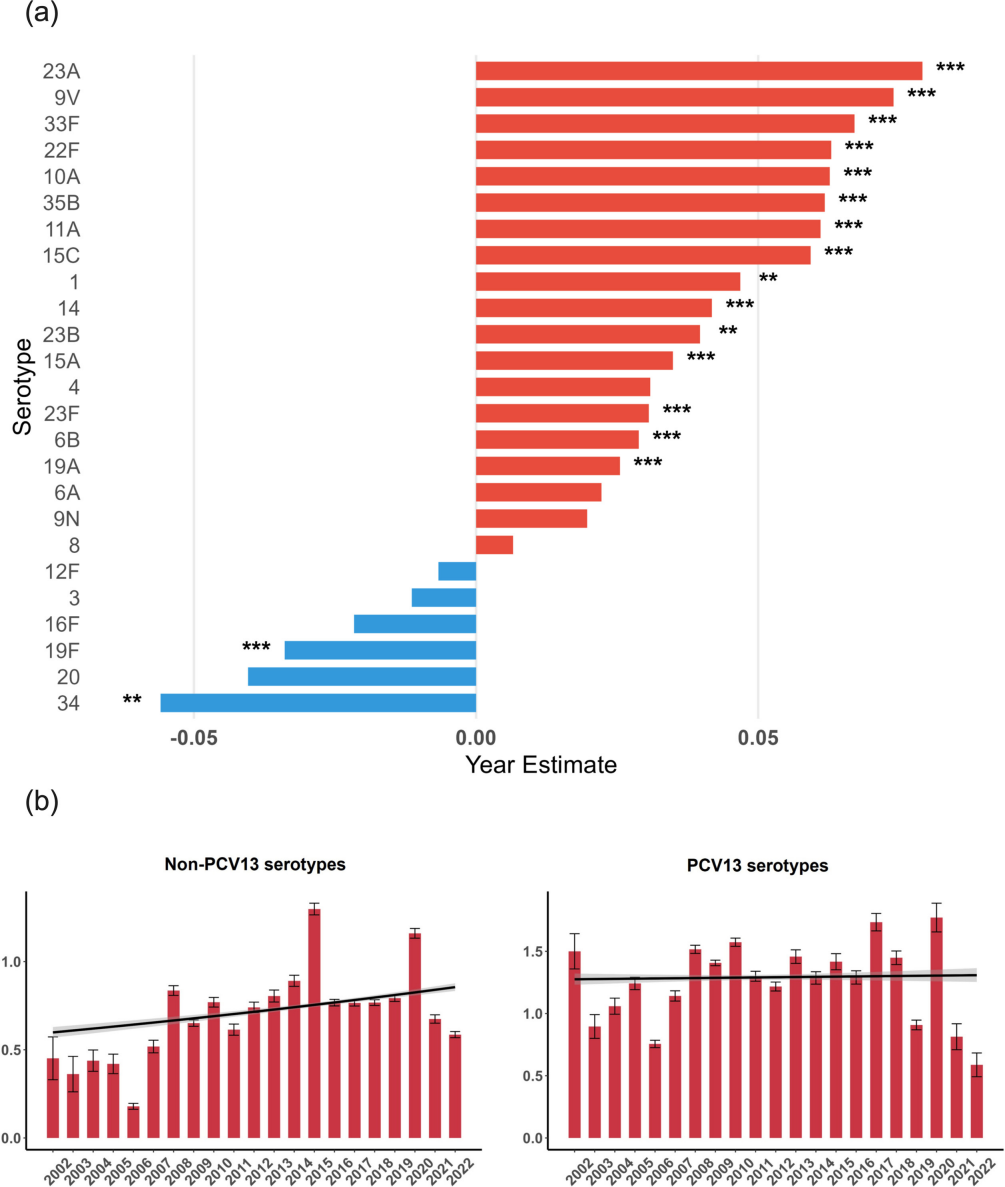
Development trends of MDR scores. (**a**) Across serotypes of *S. pneumoniae* from 2002 to 2022, with over 500 genomes available in the dataset. Bars represent estimates of yearly changes in MDR score per serotype. Asterisks indicate statistical significance by Poisson or negative binomial regression. (**b**) Across serotypes included and not included in PCV13. Bars represent the mean MDR scores with sem, while lines indicate the fitted trends from negative binomial regression, with shading representing the 95% confidence intervals. For non-PCV13 serotypes, the estimated yearly change in MDR score observed was 0.0178 (***), while for PCV13 serotypes it was 0.0012 (ns).

In total, 40,005 sequences had their serotypes predicted. Analysis of the most common serotype and their distribution over time is shown in Fig. S2. The five serotypes with a higher proportion of MDR were 19F (63.2%), 15A (43.3%), 23F (43.3%), 6B (42.5%) and 19A (34.8%). A list of the 25 most prevalent serotypes with genome counts and MDR rates is available in Table S6. When looking at the distribution of the MDR score over time, several serotypes presented different trends of resistance determinants accumulation (Fig. 6a). The higher significant increases were seen in 33F, followed by 22F, 10A, 23A and 35B. In comparison, serotypes 34, 19F and 16F showed declining patterns. Individual graphs for each serotype are shown in Fig. S3. Fig. S4 shows relative predicted resistance to each serotype to the most common antibiotic classes. Among serotypes not included in any vaccine formulation, serotype 13 presented the highest MDR percentage at 11.7%, followed by 6C at 7.5% and 7C at 6.3% (Table S7). When comparing serotypes included or not in PCV13, we observed a sharper increase in the accumulation of resistance determinants in non-vaccine serotypes (Fig. 6b). This was also observed for all the other vaccine formulations available (data not shown).

## Discussion

Analyses including data from over 75,000 genomes showed an average of just under one reported resistance determinant per pneumococcal genome, coupled with MDR being identified for 16.7% of genomes. Stable numbers of MDR were observed globally over the years; however, data from genomes collected from Asia and South America indicate strongly increasing trends in MDR. Of particular concern was the increase in macrolide resistance determinants. We utilized an *in silico* approach to predict the serotype for ~40,000 genomes and identified a high number of resistance determinants in serotypes 19F, 23F, 15A, 6B and 19A. Furthermore, upward trends of resistance presence were observed for a number of serotypes such as 33F, 22F, 10A and 23A, while decreasing trends were seen for 34, 19F and 16F. Non-vaccine serotype 13 presented a considerable MDR rate and a sharp increasing trend in ARG presence, which warrants monitoring.

Multidrug-resistant *S. pneumoniae* in young children is a significant concern [33]. We identified an overall average rate of MDR around 16%. Specific rates for each region and country varied significantly. Different study and sampling strategies make it likely that some over- and underestimations are present in the analysed dataset. However, the trend of high prevalence of MDR in Asia aligns with previous research that has reported elevated MDR rates in *S. pneumoniae* isolates, with recent data indicating rates of up to 92% [34,36]. High rates of MDR and macrolide resistance in South America have also been described previously [11,37]. The South America region has a low number of sequences collected (*n*=1,562). This is coupled with an MDR rate of over 24% and an increasing trend in recent years and highlights the necessity for increased surveillance efforts, as an increase in antibiotic usage in both South America and Asia is likely to be seen in upcoming years, together with rapid economic development in parts of these continents [38].

Macrolide resistance is a growing concern for pneumococci mediated by two main mechanisms. As macrolides target protein synthesis, *erm(B*) acts as a ribosomal methylase that dimethylates the 23S rRNA, preventing binding of the drug. Often the phenotype observed is resistance to macrolide–lincosamide–streptogramin B, referred to as the MLSB phenotype [39]. On the other hand, *mef(A*) and *mef(E*) work as an efflux pump in tandem with *msr(D*) by removing the drug from the intracellular environment. This M phenotype confers resistance to 14- and 15-membered macrolides but is sensitive to clindamycin, in contrast with the MLSB phenotype [40]. Distribution of each ARG varies, and the widespread presence of genomes containing both genes has been highlighted as a concern [12,41,44]. Our study identified *erm(B*) as the most dominant gene responsible for macrolide resistance in Asia, Europe and Oceania, whereas *mef(A*)/*msr(D*) was most prevalent in North America and in Africa. These patterns have been observed previously in a variety of studies (for a review see [12,41]). We also show that the rise in macrolide resistance is seemingly driven by *mef(A)/msr(D*). A significant proportion of dual-genotype [*erm(B*) and *mef(A*)/*msr(D*)] macrolide resistance was noted in Asia and South America, with rates over 20%, while in other regions, rates ranged between 1 and 5%. Isolates with dual genotypes have been shown to present clinical resistance to macrolides similar to isolates presenting only *erm(B*) [43]. Therefore, while the presence of two redundant determinants does not seem to lead to a higher level of macrolide resistance, these isolates have been often associated with more MDR phenotypes. We observed in our study that 98% of dual-genotype isolates were also MDR and 97.3% were predicted to be resistant to beta-lactams, in comparison to 88.7%/84.9% in pure *erm(B*) isolates and 49.1%/69.7% in *mef(A*) and *msr(D*) isolates. A plausible explanation for the finding could be attributed to the presence of multiple mobile genetic elements in dual-genotype isolates. The *erm(B*) gene is often associated with integrative and conjugative elements (ICEs) such as Tn*6002*, Tn*6003*, Tn*6009* and Tn*1545* that often co-carry other ARGs such as *tet(M*) and *aphA-3* [45,47]. Additionally, the mega element in *S. pneumoniae* carries the *mef(E*) variant of *mef(A*), and it is often also present with other resistance genes in Tn*2009*, Tn*2010* and Tn*2017* [48,50]. Together, the presence of two possible ICEs coupled with the high proportion of penicillin-binding proteins in the species increases the chance of MDR frequency in these isolates.

The two most common serotypes observed in this study were 19F and 19A, and both were associated with high rates of MDR. These serotypes are often involved with invasive pneumococcal disease (IPD) in children and adults [51,59]. However, their involvement with IPD has decreased post-PCV introduction [58,60]. From 2002 to 2022, we observed a decrease in resistance proportion in 19F isolates, which coincides with its decrease in involvement with IPD globally. On the other hand, serotype 19A emerged after the introduction of PCV7 [61]. Following the inclusion in PCV13, its rates of involvement with IPD disease have gone down [8,62]. In Belgium, after switching from PCV13 to PCV10, a rapid re-emergence of serotype 19A causing IPD, particularly in children, was observed [63]. Furthermore, it has been shown that serotypes 19A, 3 and 7C persist as nasopharyngeal colonizers even post-introduction of PCV13 [64]. Looking at development over time, an upward trend was observed for serotype 19A between 2002 and 2022, mainly driven by the period pre-PCV13. For serotype 3, another colonizer with high virulence traits (for review see [65]), our results indicate stable levels of determinants over the years, but an increase in the proportion of genomes containing *tet(32*), a tetracycline resistance gene, calls for attention and should be further monitored moving forward. The successive accumulation of ARGs even in serotypes that are covered by vaccine strategies is a concern given how prolific streptococci are at exchanging genes via horizontal gene transfer.

Upward trends of ARG/divergent allele presence were observed for a variety of non-vaccine serotypes, such as 23A and 23B. While these strains are generally more often associated with carriage and less associated with IPD [66,67], the emergence of resistance is a cause of concern, particularly for populations at higher risk of pneumococcal disease. Cases of disease for 23A and 23B have also been observed more frequently after the introduction of PCV13 [62], as well as higher levels of resistance [68,69]. 23A and 23B have only been included in the recent PCV21, which tries to tackle problematic serotypes in adults. Serotype 13 is not included in vaccine formulations currently and has shown a significant rise in resistance determinants recently. The introduction of PCVs has clearly reduced pneumococcal disease rates globally [5] and has had positive general effects on AMR [70]. However, due to limitations in the number of serotypes that are covered in the PCVs, their implementation has led to significant changes in serotype distribution, with non-vaccine types increasing in proportion [71,72]. Understanding these influences is central for interpreting the relationship between vaccine implementation and serotype diversity and will continue to be crucial to follow up on population dynamics moving forward.

While this study provides a global overview of AMR in *S. pneumoniae* isolates, it is important to acknowledge several limitations in the dataset. Data available in public repositories are not the result of systematic random sampling and can present bias resulting in over- or under-presenting AMR. In particular, such selection bias has potentially influenced the data collected in countries with fewer isolates, as it is possible that the focus for sequencing efforts was either hypervirulent and/or non-susceptible isolates. This is also likely to be more evidently pronounced in older time points due to high costs and time of sequencing. Nevertheless, as data increased and gained robustness in more recent years, this risk has been partially mitigated. Thus, confidence is added to the increasing trends we observed in recent years, despite the potential bias of over-representation in more distant time points.

Generally, recent studies have shown a high level of accuracy regarding *in silico* prediction of AMR [30,73, 74] and serotype [75,78]. In addition, it is likely that a proportion of the predicted resistance determinants will be associated with no or intermediate resistance, e.g. divergent *pbps* might not necessarily lead to consistent and predictable beta-lactam resistance. In such instances, mosaic sequences and combinations of *pbp* mutations are challenging to predict and classify [79], including also the involvement of additional genetic elements such as the *murMN* operon that encodes enzymes involved in cell wall synthesis [80]. The combination of such factors may lead to various levels of penicillin resistance and extended-spectrum cephalosporin resistance [81,82]. In our analyses, a decrease in divergent *pbp* proportion was observed, so it is unlikely that possible overestimations of predicted *pbp* phenotypes have impacted trend analyses for MDR and serotypes. It is important to highlight, however, that further research on this topic, including sequences from closely related commensal species, is warranted to better unravel the puzzle of beta-lactam resistance in streptococci from the mitis group. Nevertheless, timely detection of increased spread of resistance determinants can offer an early warning system to surveillance authorities and guide future research.

The sharing and reusability potential of genomic data is a priority highlighted by a number of stakeholders worldwide [18,83, 84]. An important advantage of WGS monitoring is the possibility of linkage with metadata. This allows for the comparison across collection sites coupled with the possibility of correlation with clinical characteristics. Thus, poorly curated or lack of metadata significantly impairs the potential for such analyses. Multivariable analyses that would allow for a better assessment of explanatory factors underlying the changes observed are also hindered by these obstacles. In the data utilized in this study, over a third of genomes lacked information on time of collection, location and source. With better standard reporting practices, the association among different isolates, serotypes and forms of disease could be better addressed in future studies. These aspects are likely to play a major role moving forward as next generation sequencing become more affordable and the collective amount of sequencing data rapidly increases. However, to fully explore the potential of public repositories for genomic data, it is crucial that better standardization pipelines for depositing genomic data are adopted [85,86].

This study provides valuable insights for tracking pneumococcal resistance trends and highlights the importance of continuous surveillance. Such findings can assist in guiding the development of effective policies aimed at managing pneumococcal resistance and ensuring appropriate interventions. We highlight variations in the presence of resistance determinants globally as well as across serotypes over time in *S. pneumoniae*. Collectively, these data underscore the added value of utilizing public data to gain knowledge on the effectiveness and repercussions of treatment and vaccination strategies.

## Supplementary material

10.1099/mgen.0.001444 Uncited Supplementary Material 1.

10.1099/mgen.0.001444 Uncited Supplementary Material 2.

## Abbreviations

AMR: antimicrobial resistance
ARG: antimicrobial resistance gene
ICE: integrative and conjugative element
IPD: invasive pneumococcal disease
MDR: multidrug resistance
NCBI: National Center for Biotechnology Information
PCV: pneumococcal conjugate vaccine
WGS: whole-genome sequencing
