# Supplementary material for: Large-scale global molecular epidemiology of antibiotic resistance determinants in Streptococcus pneumoniae
Source: Microb Genom. 2025 Jul 2;11(7):001444. doi: 10.1099/mgen.0.001444 (PMC12282287; doi:10.1099/mgen.0.001444)
Supplement: Uncited Supplementary Material 1. [file mgen-11-01444-s001.pdf]

Supplementary material for

Large-scale global molecular epidemiology of antibiotic resistance determinants in *Streptococcus pneumoniae*

Kazi Shefaul Mulk Shawrob[1], Achal Dhariwal[1], Gabriela Salvadori[1,2], Rebecca Ashley Gladstone[3], Roger Junges[1#]

[1] *Institute of Oral Biology, Faculty of Dentistry, University of Oslo, Oslo, Norway*
[2] *The Intervention Centre, Oslo University Hospital, Oslo, Norway*
[3] *Department of Biostatistics, Faculty of Medicine, University of Oslo, Oslo, Norway*

[#]corresponding author
roger.junges@odont.uio.no
Roger Junges, DDS, PhD
Associate Professor
Institute of Oral Biology
Faculty of Dentistry, University of Oslo (UiO)
Postboks 1052 Blindern 0316 - Oslo, Norway

Supplementary material

Dataset curation and visualization

The dataset was cleaned to ensure consistency and readiness for further analysis. We extracted country name from the "Location" column, separating the country name from other details by removing everything after the first colon (":"). Terms indicating missing country data (e.g., "not collected", "not available") were identified and replaced with "Not Available" for consistency. Countries were labeled with their continents using the countrycode package (v1.6.0), distinguishing between North and South America. A "Year" column was added by extracting years from collection dates. The dplyr (v1.1.4) and stringr packages (v1.5.1) were used to extract the year from the date depending on various formats (e.g., "2006-02-26" to "2006", "2001-09" to "2001"). If the field contained only the year, that value was retained.

To extract and analyze genes marked as "=COMPLETE" from the AMR.genotypes column, a function was created to find and extract all complete genes. This function was applied to each row in the AMR.genotypes column, and the results were saved in a new column called "Complete_Genes". Rows where the "Complete_Genes" column was empty were removed. Gene names were further refined by removing text up to the next comma after an "=" sign (e.g., "*erm(A)*=COMPLETE, *pmrA*=COMPLETE" to "*erm(A)*,*pmrA*"). A matrix was created to link genes to their corresponding resistance classes (e.g., "*ant(9)-Ib*" to "AMINOGLYCOSIDE"). A custom function was defined to search through gene names, and link them with their corresponding resistance class, and store the updated information in a new "gene_to_class" column. Additionally, a custom function was implemented to identify and extract unique AMR classes for each genome, and the results were stored in the "unique_classes" column. Other variables were created based on the presence or absence of specific genes, resistance to specific classes of antibiotics, and the presence of serotypes included in specific vaccines.

Serotype classification

Initial prediction was performed with PneumoKITy [1] and PfaSTer [2]. Sequences that lacked quality or coverage were discarded from the analyses. If the predictions matched, the results were then kept; if the predictions were not a match, we attempted to call each isolate with PneumoCAT [3], which utilizes reads and quality scores enabling the tool to make calls between similar serotypes. To obtain the reads, the biosample accession numbers were tracked

through the Sequence Read Archive (SRA), and a pipeline was used to retrieve the SRA IDs, download the sequences, and convert them into fastq format for further processing using NCBI SRA Tool (v3.1.1). If no accession number was available or the fastq sequences were not able to be traced, we searched the NCBI database for information from the submitter regarding the serotyping of the deposited isolates. For serogroup 6, user submitted data and SeroBA [4] were employed to call genotype 6E into serotypes 6A or 6B.

Gene co-occurrence and data visualization

For gene co-occurrence, we filtered the dataset to include only MDR isolates. We split gene names, created a binary matrix representing gene presence/absence using Base R function, and computed a co-occurrence matrix via matrix multiplication. The resulting matrix was filtered to include only gene pairs over the threshold of 100 occurrences. In addition, we reshaped the matrix into a data frame where each row represents a gene pair and its co-occurrence frequency, and visualized as a chord diagram with circlize (v0.4.16).

Data visualization was performed using R Studio, with the ggplot2 (v 3.5.1) package used as the primary tool for generating the figures presented in this study. For geospatial analyses and world map creation, the rworldmap (v1.3.8) package was employed. Additionally, heatmaps were constructed using the ComplexHeatmap (v2.18.0) package.

References

1. Sheppard CL, Manna S, Groves N, Litt DJ, Amin-Chowdhury Z, Bertran M, et al. PneumoKITy: A fast, flexible, specific, and sensitive tool for *Streptococcus pneumoniae* serotype screening and mixed serotype detection from genome sequence data. Microbial Genomics. 2022;8(12):000904.
2. Lee JT, Li X, Hyde C, Liberator PA, Hao L. PfaSTer: a machine learning-powered serotype caller for *Streptococcus pneumoniae* genomes. Microb Genom. 2023;9(6).
3. Kapatai G, Sheppard CL, Al-Shahib A, Litt DJ, Underwood AP, Harrison TG, et al. Whole genome sequencing of *Streptococcus pneumoniae*: development, evaluation and verification of targets for serogroup and serotype prediction using an automated pipeline. PeerJ. 2016;4:e2477.
4. Epping L, van Tonder AJ, Gladstone RA, The Global Pneumococcal Sequencing C, Bentley SD, Page AJ, et al. SeroBA: rapid high-throughput serotyping of *Streptococcus pneumoniae* from whole genome sequence data. Microb Genom. 2018;4(7).

Table S2. Serotypes included in each pneumococcal vaccine.

Vaccine	Serotypes
PCV7	4, 6B, 9V, 14, 18C, 19F, 23F
PCV10	4, 6B, 9V, 14, 18C, 19F, 23F, 1, 5, 7F
PCV13	4, 6B, 9V, 14, 18C, 19F, 23F, 1, 5, 7F, 3, 6A, 19A
PCV15	4, 6B, 9V, 14, 18C, 19F, 23F, 1, 5, 7F, 3, 6A, 19A, 22F, 33F
PCV20	4, 6B, 9V, 14, 18C, 19F, 23F, 1, 5, 7F, 3, 6A, 19A, 22F, 33F, 15B, 12F, 11A, 10A, 8
PPSV23	4, 6B, 9V, 14, 18C, 19F, 23F, 1, 5, 7F, 3, 19A, 22F, 33F, 15B, 12F, 11A, 10A, 8, 20, 17F, 9N, 2
PCV21	1, 3, 4, 5, 6A, 6B, 7F, 9V, 11A, 14, 15A, 15C, 16F, 18C, 19A, 19F, 23A, 23B, 23F, 24F, 31, 35B

Table S3. Distribution of isolates collected by country with MDR score data.

Country	n	%	Mean MDR score	SEM
Argentina	2	.0	2.00	1.00
Australia	1657	2.2	0.55	0.02
Austria	1	.0	3.00	
Bangladesh	94	.1	1.21	0.10
Belarus	72	.1	1.82	0.17
Belgium	213	.3	1.03	0.11
Botswana	1	.0	0.00	
Brazil	508	.7	1.05	0.05
Burkina Faso	1	.0	2.00	
Cambodia	56	.1	2.14	0.11
Cameroon	8	.0	1.75	0.37
Canada	1854	2.5	0.56	0.02
Central African Republic	4	.0	2.00	0.41
China	3166	4.2	2.75	0.01
Colombia	6	.0	1.50	0.67
Croatia	6	.0	1.50	0.34
Czechia	43	.1	0.70	0.14
Denmark	57	.1	0.46	0.13
Egypt	34	.0	1.91	0.18
Ethiopia	43	.1	1.74	0.22
Finland	1	.0	0.00	
France	40	.1	2.28	0.28
Gambia	1638	2.2	0.68	0.02
Germany	330	.4	1.10	0.05
Ghana	55	.1	1.78	0.11
Greece	2	.0	2.50	0.50
Greenland	2	.0	0.00	0.00
Hong Kong	55	.1	1.96	0.07
Hungary	24	.0	0.83	0.26
Iceland	120	.2	3.74	0.05
India	964	1.3	1.60	0.04
Indonesia	92	.1	1.73	0.12
Ireland	152	.2	0.57	0.08
Israel	1148	1.5	0.91	0.03
Italy	14	.0	1.43	0.25
Japan	220	.3	2.61	0.05
Kazakhstan	1	.0	1.00	
Kenya	1	.0	1.00	

Kuwait	1	.0	3.00	
Lebanon	9	.0	2.78	0.22
Lithuania	1	.0	0.00	
Malawi	4238	5.6	1.04	0.02
Malaysia	47	.1	1.85	0.22
Mexico	14	.0	3.14	0.10
Mongolia	1	.0	1.00	
Morocco	42	.1	1.26	0.15
Mozambique	168	.2	0.92	0.06
Myanmar	58	.1	1.60	0.15
Nepal	416	.6	0.79	0.05
Netherlands	1775	2.4	0.23	0.01
New Zealand	718	1.0	0.60	0.03
Niger	15	.0	1.27	0.23
Norway	24	.0	0.00	0.00
Oman	1	.0	4.00	
Papua New Guinea	2	.0	1.00	0.00
Peru	1043	1.4	1.51	0.04
Poland	284	.4	2.09	0.09
Portugal	192	.3	1.08	0.11
Qatar	95	.1	1.40	0.11
Russia	143	.2	1.12	0.11
Senegal	31	.0	0.94	0.19
Singapore	2	.0	1.50	1.50
Slovenia	91	.1	0.98	0.13
South Africa	5037	6.7	0.92	0.01
South Korea	45	.1	3.00	0.10
Spain	241	.3	1.29	0.08
Sweden	64	.1	0.14	0.07
Switzerland	11	.0	0.36	0.24
Taiwan	94	.1	3.31	0.05
Tanzania	26	.0	2.04	0.23
Thailand	3072	4.1	1.65	0.02
Togo	20	.0	1.35	0.17
Trinidad and Tobago	84	.1	0.75	0.11
Turkey	15	.0	2.27	0.33
United Kingdom	1292	1.7	0.29	0.02
United States	21488	28.6	0.71	0.01
Uruguay	3	.0	3.00	0.00
Vietnam	18	.0	2.89	0.21

Table S4. Antibiotic resistance genes (ARGs) identified in *S. pneumoniae* isolates.

Antibiotic class	Gene	Count	Relative to ARGs	Relative to genomes
Aminoglycoside	*ant(2")-Ia*	1	0.00	0.00
	ant(6)-Ia	4	0.00	0.01
	ant(9)-Ib	4	0.00	0.01
	aph(3')-Ia	1	0.00	0.00
	aph(3')-IIIa	315	0.26	0.42
	str	18	0.01	0.02
	aac(6')-Ie/aph(2")-Ia	14	0.01	0.02
	aadD1	2	0.00	0.00
Beta-lactam	*bla2*	1	0.00	0.00
	blaARL	3	0.00	0.00
	blaARL-2	11	0.01	0.01
	blaI	20	0.02	0.03
	blaPC1	11	0.01	0.01
	blaR1	23	0.02	0.03
	blaTEM	3	0.00	0.00
	blaTEM-1	1	0.00	0.00
	blaTEM-116	4	0.00	0.01
	blaTEM-135	1	0.00	0.00
	blaTEM-171	1	0.00	0.00
	blaZ	13	0.01	0.02
	mecA	17	0.01	0.02
	mecA1	5	0.00	0.01
	mecI	2	0.00	0.00
	mecR1	2	0.00	0.00
	divergent-*pbp1a*s	14436	11.77	19.21
	divergent-*pbp2b*s	25690	20.94	34.18
	divergent-*pbp2x*s	24183	19.71	32.17
Bleomycin	*bleO*	1	0.00	0.00
	ble-Sh	1	0.00	0.00
Fosfomycin	*fosB*	5	0.00	0.01
	fosY	1	0.00	0.00
Fusidane	*fusD*	10	0.01	0.01
Glycopeptide	*vanC1*	1	0.00	0.00
	vanG	1	0.00	0.00
	vanR-C	1	0.00	0.00
	vanS-C	1	0.00	0.00
	vanT-C	1	0.00	0.00
	vanXY	1	0.00	0.00
	vanXY-C	1	0.00	0.00
Lincosamide	*lnu(A)*	35	0.03	0.05
	lnu(A)'	3	0.00	0.00
	lsa(C)	10	0.01	0.01

	sal(A)	1	0.00	0.00
	vga(A)-LC	5	0.00	0.01
Macrolide	*erm(A)*	4	0.00	0.01
	erm(B)	10525	8.58	14.00
	erm(C)	41	0.03	0.05
	erm(X)	3	0.00	0.00
	mef(A)	12032	9.81	16.01
	mph(C)	11	0.01	0.01
	msr(A)	5	0.00	0.01
	msr(D)	12020	9.80	15.99
Phenicol	*catA*	2833	2.31	3.77
	catA1	3	0.00	0.00
	catA16	145	0.12	0.19
	catP	1	0.00	0.00
	cmx	4	0.00	0.01
Streptothricin	*sat4*	209	0.17	0.28
Sulfonamide	*sul1*	3	0.00	0.00
Tetracycline	*tet(32)*	160	0.13	0.21
	tet(38)	2	0.00	0.00
	tet(K)	132	0.11	0.18
	tet(L)	16	0.01	0.02
	tet(M)	19429	15.84	25.85
	tet(O)	33	0.03	0.04
	tet(S)	150	0.12	0.20
	tet(W)	2	0.00	0.00
	tetA(60)	1	0.00	0.00
	tetB(60)	1	0.00	0.00
Trimethoprim	*dfrE*	3	0.00	0.00
	dfrG	28	0.02	0.04
	dfrS1	12	0.01	0.02
**Total**		**122673**		

Table S5. Distribution of macrolide resistance in *S. pneumoniae* determined by *erm(B)* and/or *mef(A)/msr(D)* across continents between 2015-2022.

	mef(A)		*erm(B)*		Dual genotype		Total genomes
	n	%	n	%	n	%	
Africa	482	16.07 %	37	1.23 %	13	0.43 %	3000
Asia	652	35.45 %	1451	78.90 %	456	24.80 %	1839
Europe	70	6.36 %	158	14.36 %	49	4.46 %	1102
North America	3997	21.49 %	1826	9.82 %	331	1.78 %	18595
South America	155	40.90 %	161	42.48 %	77	20.32 %	379
Oceania	52	3.40 %	136	8.89 %	19	1.24 %	1529
Total	5409	20.42 %	3786	14.29 %	945	3.58 %	26495

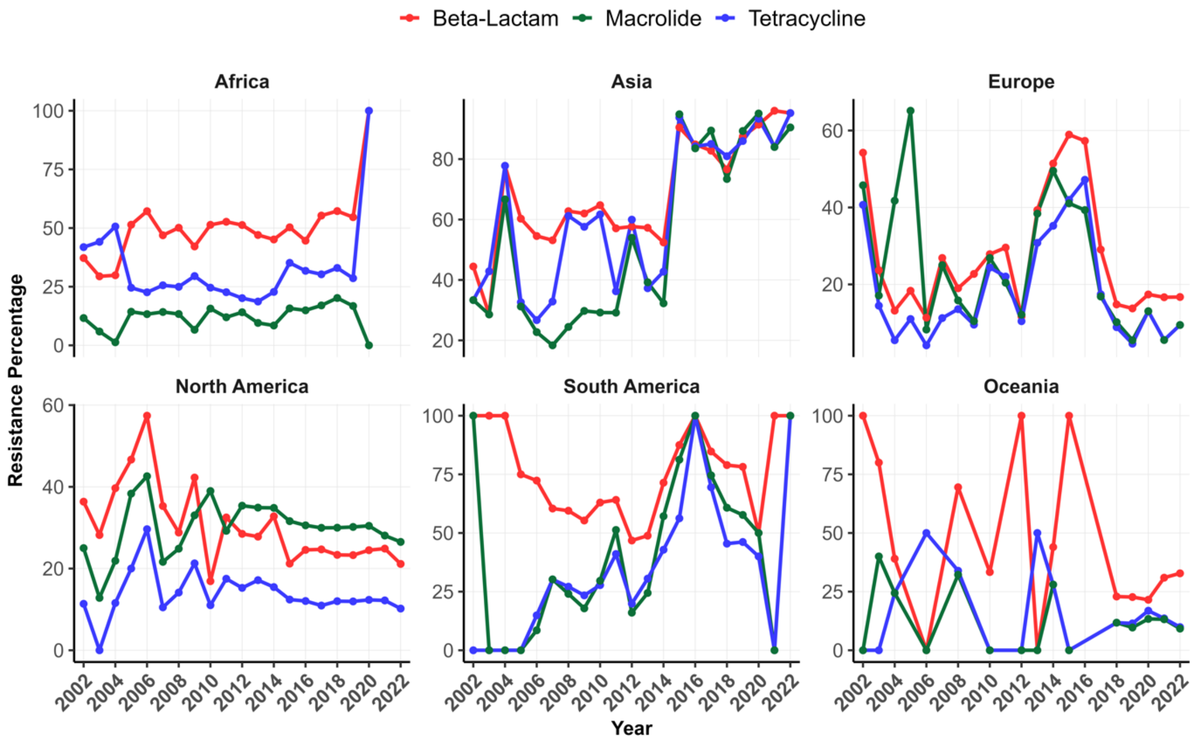

Fig S1. Distribution of resistance to the three most common antibiotic classes (beta-lactams, macrolides, and tetracyclines) over time. Each segment of the panel represents a continent.

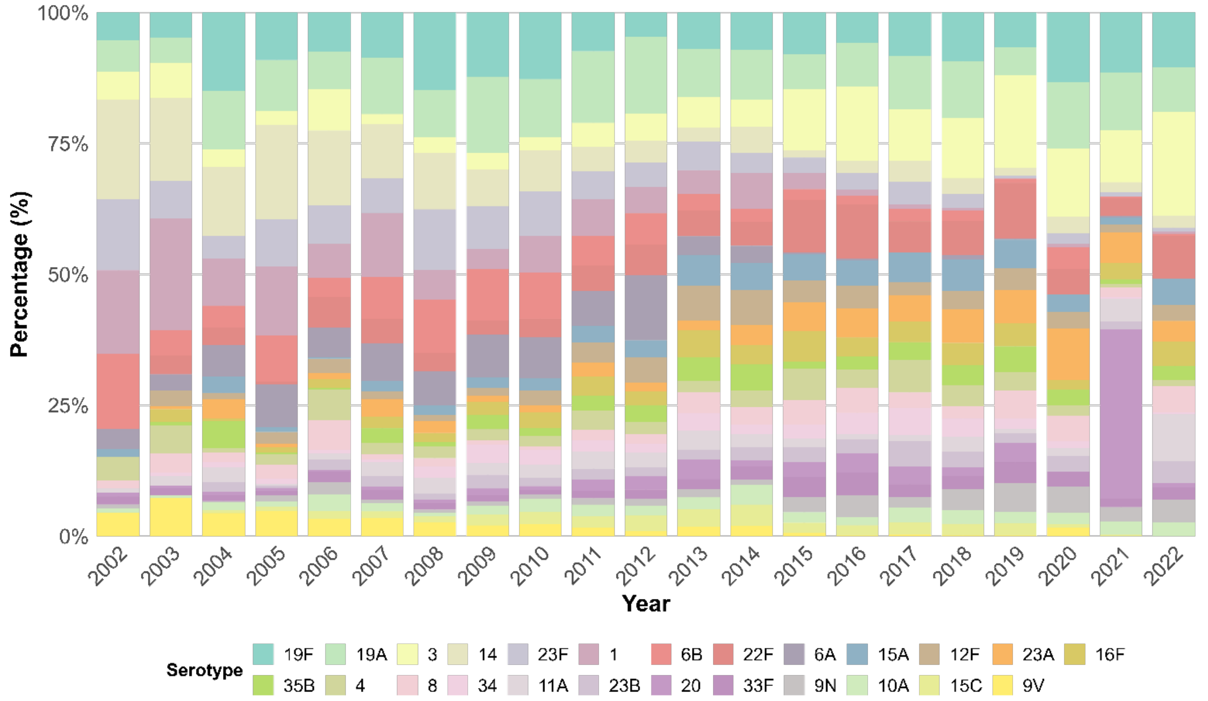

Fig S2. Stacked bar chart showing the serotype composition each year for the 25 most prevalent.

Table S6. Distribution and MDR rate of serotypes with more than 100 genomes available and their trends of development. Serotypes whose trend was not statistically significant were considered stable. **p<0.01***p<.001

Serotype	Number of genomes	% of MDR	Year estimate	Trend	Statistically significant
**19F**	3792	63.2	-0.03	Down	***
**19A**	3224	34.8	0,03	Up	***
**3**	2604	8.1	-0,01	Stable	---
**14**	2160	24.4	0.04	Up	***
**23F**	2036	43.3	0.03	Up	***
**1**	1837	0.8	0.05	Up	**
**6B**	1821	42.5	0.03	Up	***
**22F**	1724	1.6	0.06	Up	***
**6A**	1533	5.6	0.02	Stable	---
**15A**	1043	43.3	0.03	Up	***
**12F**	1028	8.6	-0.01	Stable	---
**23A**	1012	28.4	0.08	Up	***
**16F**	948	2.5	-0.02	Stable	---
**35B**	909	1.8	0.06	Up	***
**4**	893	1.6	0.03	Stable	---
**8**	770	0.4	0.01	Stable	---
**34**	701	3.1	-0.06	Down	**
**11A**	670	0.9	0.06	Up	***
**20**	638	2	-0.04	Stable	---
**23B**	638	0.8	0.04	Up	**
**33F**	637	1.9	0.07	Up	***
**9N**	613	0.3	0.02	Stable	---
**10A**	595	4.5	0.06	Up	***
**15C**	598	12.9	0.06	Up	***
**9V**	559	7.9	0.07	Up	***
**5**	485	1.9	0.01	Stable	---
**13**	480	11.7	0.13	Up	***
**21**	447	2.7	-0.15	Down	***
**35F**	445	0.4	0.01	Stable	---
**17F**	434	3.9	0.08	Up	***
**15B**	426	6.9	0.00	Stable	---
**6C**	412	7.5	0.02	Stable	---
**7C**	382	6.3	-0.03	Stable	---
**7A**	378	0.5	0.03	Stable	---
**31**	335	0.9	0.05	Stable	---
**18C**	300	0.67	0.07	Stable	---
**38**	295	2.4	-0.14	Down	**
**7F**	242	0	0.09	Stable	---
**6D**	194	3.7	0.11	Up	***
**10B**	167	2.9	0.13	Up	***
**18A**	112	3.6	0.02	Stable	---

19F

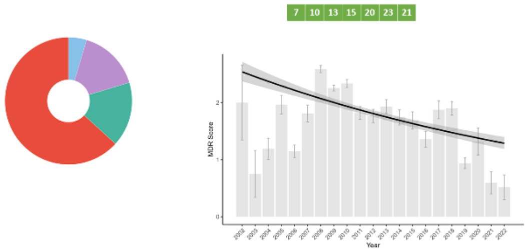

19A

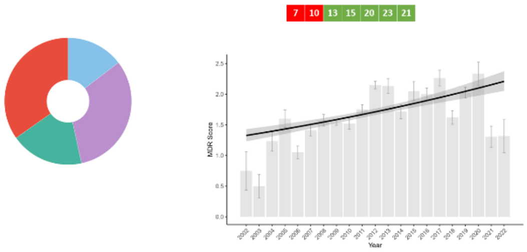

3

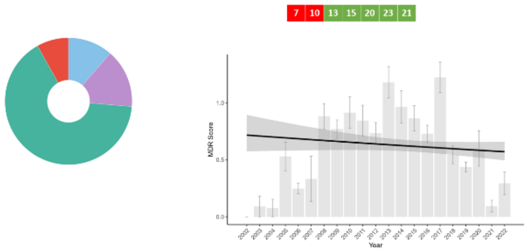

14

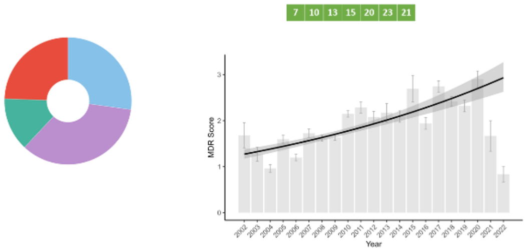

23F

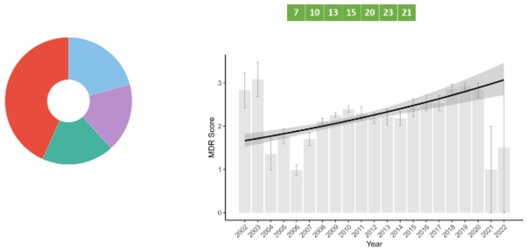

10A

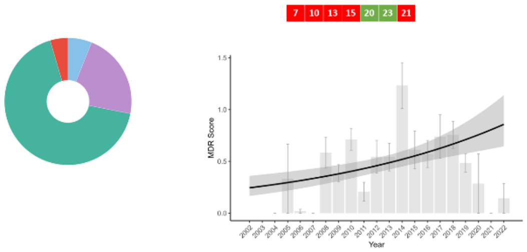

6B

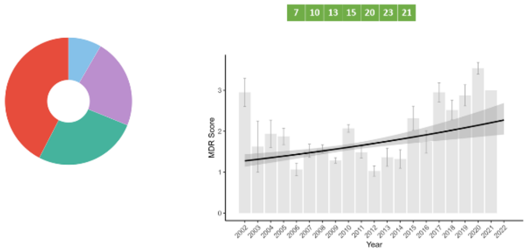

22F

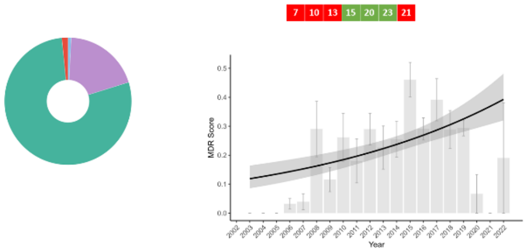

6A

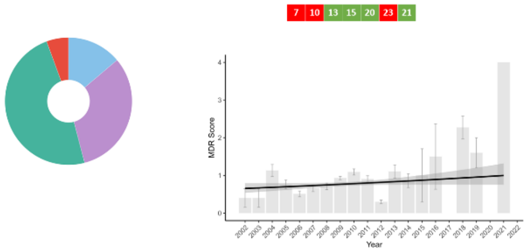

15A

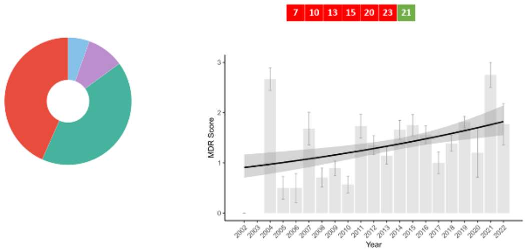

12F

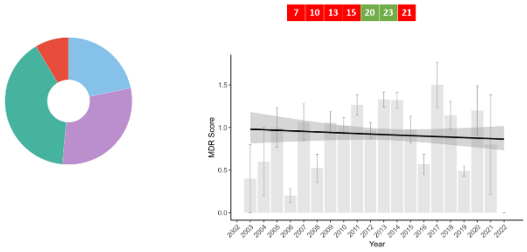

23A

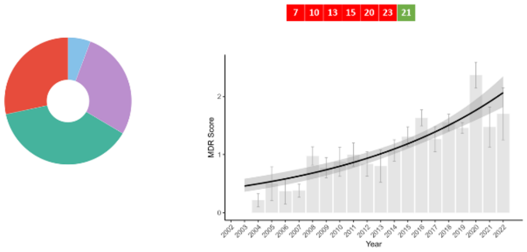

16F

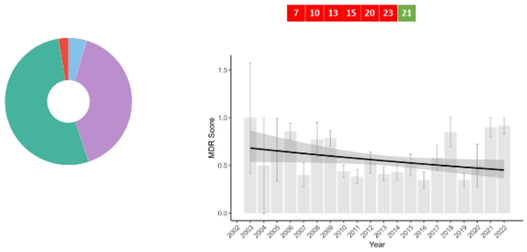

35B

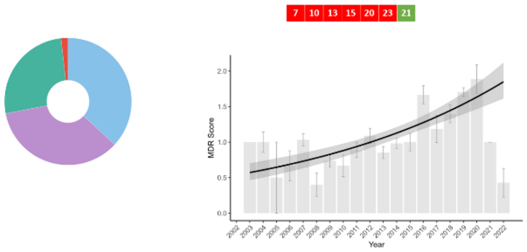

4

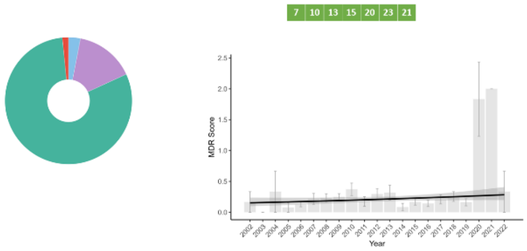

8

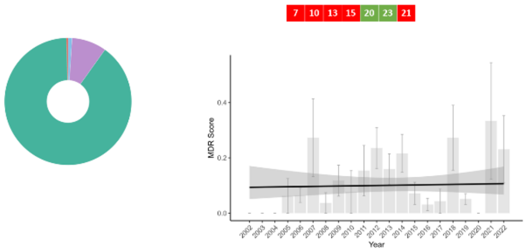

34

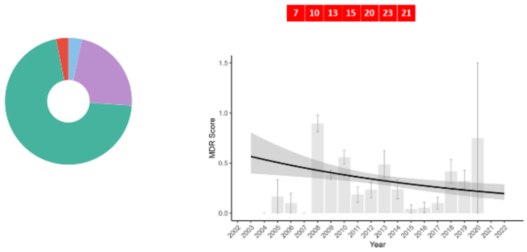

11A

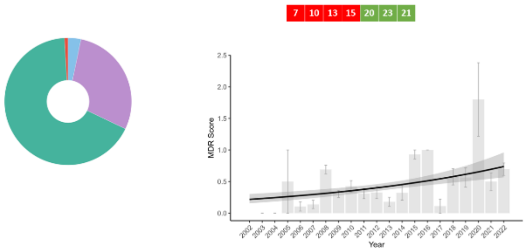

20

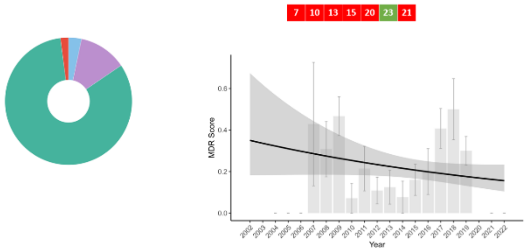

23B

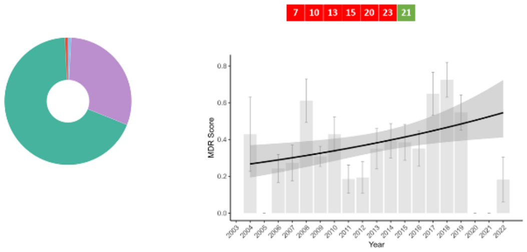

33F

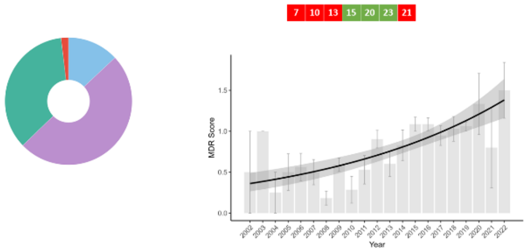

9N

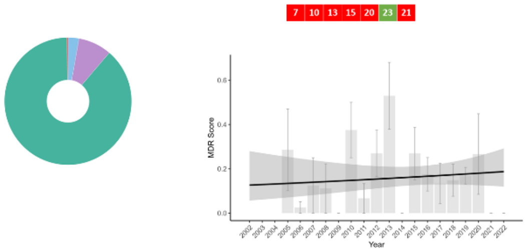

10A

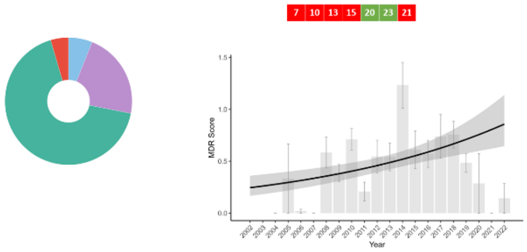

15C

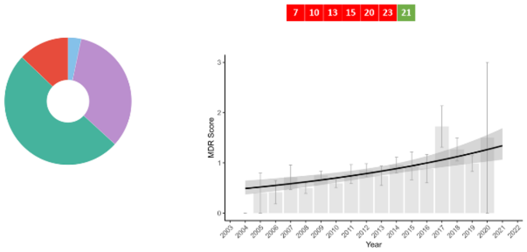

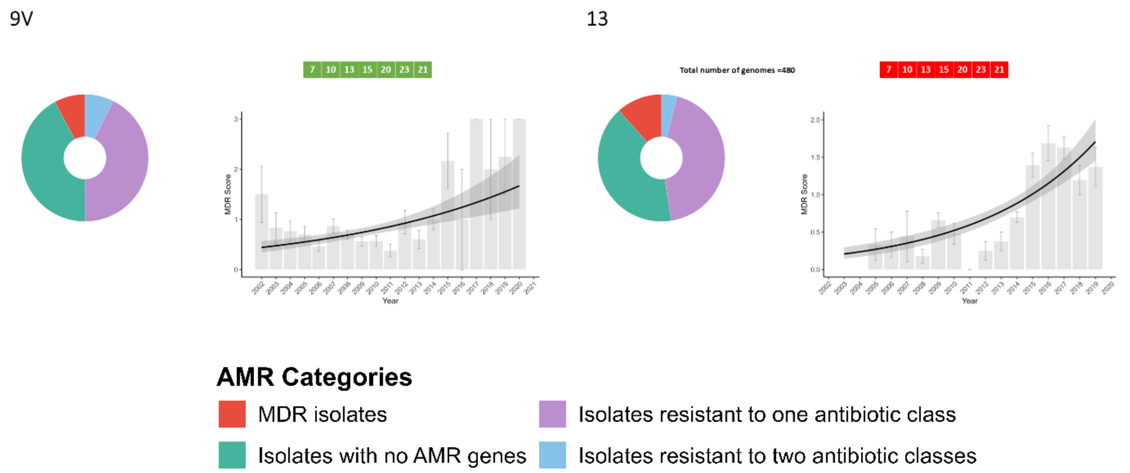

Fig S3. Trends over time for individual serotypes and fitted lines for negative binomial regression. Red sections in the donut plot indicate MDR, while green indicates resistance to 2 antibiotic classes, and purple indicates resistance to 1 antibiotic class. Graphs to the right show average MDR score with SEM for every year from 2002 to 2022. Green blocks in the top right indicate inclusion in the vaccine whereas red blocks indicate non-inclusion.

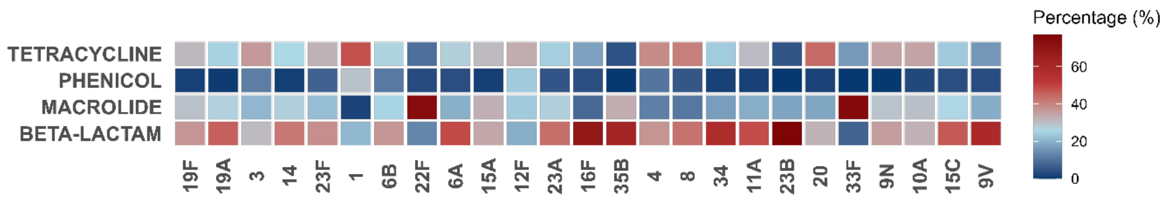

Fig S4. Reported resistance to the four most common antibiotic classes in the 25 serotypes with most genomes. Proportions are shown as the percentage of serotype resistance to the corresponding antibiotic class.

Table S7. MDR rate of serotypes with over 100 genomes that have not been included in any vaccine formulations.

Serotype	MDR	not MDR	Total	MDR%
**34**	22	679	701	3.1
**13**	56	424	480	11.7
**21**	12	435	447	2.7
**35F**	2	443	445	0.4
**6C**	31	382	413	7.5
**7C**	24	358	382	6.3
**7A**	2	376	378	0.5
**38**	7	288	295	2.4
**6D**	7	187	194	3.6
**10B**	5	162	167	3
**18A**	4	108	112	3.6
**22A**	3	102	105	2.9
